# Chorionicity-associated variation in metabolic phenotype of cord blood in twin

**DOI:** 10.1186/s12986-023-00744-1

**Published:** 2023-07-13

**Authors:** Xiaoyu Liu, Jing Yang, Rui Ran, Fei Long, Yang Yang, Xiaojing Dong, Richard Saffery, Boris Novakovic, Hatem Mousa, Yuan Wei, Lina Hu, Ting-Li Han

**Affiliations:** 1 Department of Obstetrics and Gynecology, Second Affiliated Hospital of Chongqing Medical University, Chongqing, China; 2 Department of Obstetrics and Gynecology, Peking University Third Hospital, Beijing, China; 3 State Key Laboratory of Ultrasound Engineering in Medicine Co-Founded by Chongqing and the Ministry of Science and Technology, School of Biomedical Engineering, Chongqing Medical University, Chongqing, China; 4 Department of Obstetrics and Gynecology, First Affiliated Hospital of Chongqing Medical University, Chongqing, China; 5 Molecular Immunity, Murdoch Children’s Research Institute, Melbourne, VIC Australia; 6 Department of Paediatrics, University of Melbourne, Melbourne, VIC Australia; 7 University of Leicester, NHS Trust, Leicester, UK; 8 Mass Spectrometry Centre of Maternal Fetal Medicine, Life Science Institution, Chongqing Medical University, Chongqing, China

**Keywords:** Twin pregnancies, Umbilical cord plasma, Monochorionic, MC, Dichorionic, DC, Metabolomics

## Abstract

**Background:**

Monochorionic (MC) twins present a higher incidence of unfavorable clinical perinatal outcomes than dichorionic (DC) twins, often in association with placental vascular anastomosis. In this study, we profiled the umbilical cord plasma metabolomes of uncomplicated MC and DC twin pregnancies and related these to several offspring outcomes, previously associated with birthweight.

**Methods:**

Umbilical vein blood samples were collected at birth from 25 pairs of uncomplicated MC twins and 24 pairs of uncomplicated DC twins. The samples were subjected to gas chromatography-mass spectrometry-based metabolomics. 152 metabolites were identified from the cord plasma samples of MC and DC twins. Partial least squares discriminant analysis and pathway analysis were performed to compare within DC/MC twin pairs and between DC and MC twins. A generalized estimating equation (GEE) model was utilized to explore the correlation between metabolic differences and birthweight discordance within and between twin pairs.

**Results:**

Our study revealed clear differences between the metabolite profiles of umbilical cord plasma of MC and DC twins. Metabolite profiles in MC within twin pairs and DC within twin pairs were characterized by the differences in 2 − hydroxyglutaramic acid levels and nicotinamide levels, respectively. The metabolic pathways of GSH, tryptophan, and fatty acid metabolism, were significantly downregulated in MC twins compared to DC twins. In addition, the concentration of caffeine and decamethyl-cyclopentasiloxane (D5) was positively correlated with birthweight in MC and DC twins.

**Conclusion:**

This study demonstrated that the altered metabolites in umbilical plasma made contributions to the different chorionicities between uncomplicated MC twins and DC twins. The chorionicity of twins seems to affect the metabolic cross-talk between co-twin pairs and be related to birthweight discordance of twins.

**Supplementary Information:**

The online version contains supplementary material available at 10.1186/s12986-023-00744-1.

## Background

In recent decades, advanced maternal age and the increasing use of assisted reproductive technologies have resulted in a significant increase in multiple births [1]. Twin pregnancies accounted for 0.78% of all births in China in 1989 [2] but more than doubled to 1.88% from 2007 to 2014 [3]. Twin pregnancies are associated with a higher incidence of maternal and fetal complications [4, 5] compared to singletons. Rates of stillbirth and neonatal mortality for twin gestations are 14.1 and 26.1 per 1000 total births, respectively [3]. Compared with singleton pregnancies, monochorionic (MC) twin pregnancies showed a thirteenfold increase, while dichorionic (DC) twins showed a fivefold increase, in rates of stillbirth [6–8]. MC twins have a significantly greater incidence of perinatal death (11.6% in MC twins versus 5.0% in DC twins), necrotising enterocolitis (OR 4.05, 95% CI 1.97–8.35), and neurological injury compared to DC twins [9]. Unequal sharing of the placental territory and vascular communications between twins made a remarkable contribution to differentiating fetal development [9, 10]. Although multiple studies have aimed to address the issue of clinical treatment and delivery outcomes of twins through retrospective analysis [11–13], the underlying metabolic differences in twin pregnancies, associated with adverse perinatal outcomes, have not been explored in detail. This is particularly true for MC relative to DC twin pairs.

Metabolomics enables the investigation of both normal physiology and the pathophysiology of many diseases by using advanced analytical chemistry techniques [14]. Identification of specific metabolites associated with twin pregnancies generally, or distinct to MC relative to DC pregnancies, using global untargeted approaches, has the potential to provide insights into clinical management. A growing number of studies have attempted to identify metabolic variations associated with the pathophysiology of MC-specific complications [15–17] and most have found disrupted amino acid and fatty acid metabolism in umbilical cord blood and/or placental tissue [15, 16]. However, these studies failed to investigate the metabolism of uncomplicated DC twin pregnancies and to differentiate between DC and MC pairs. Comparing uncomplicated MC twins with DC twins provide a favorable comparative study to investigate the effect of chorionicity between siblings on placental metabolite allocation.

Here, we hypothesized that the umbilical cord blood of MC and DC twins from uncomplicated pregnancies would show differing metabolite profiles, potentially in association with placenta-specific factors. Thus, the present study aimed to enrich the understanding of chorionicity and enable comparison with pathological twin gestations in future studies.

## Methods

### Study design

**Fig. 1:**
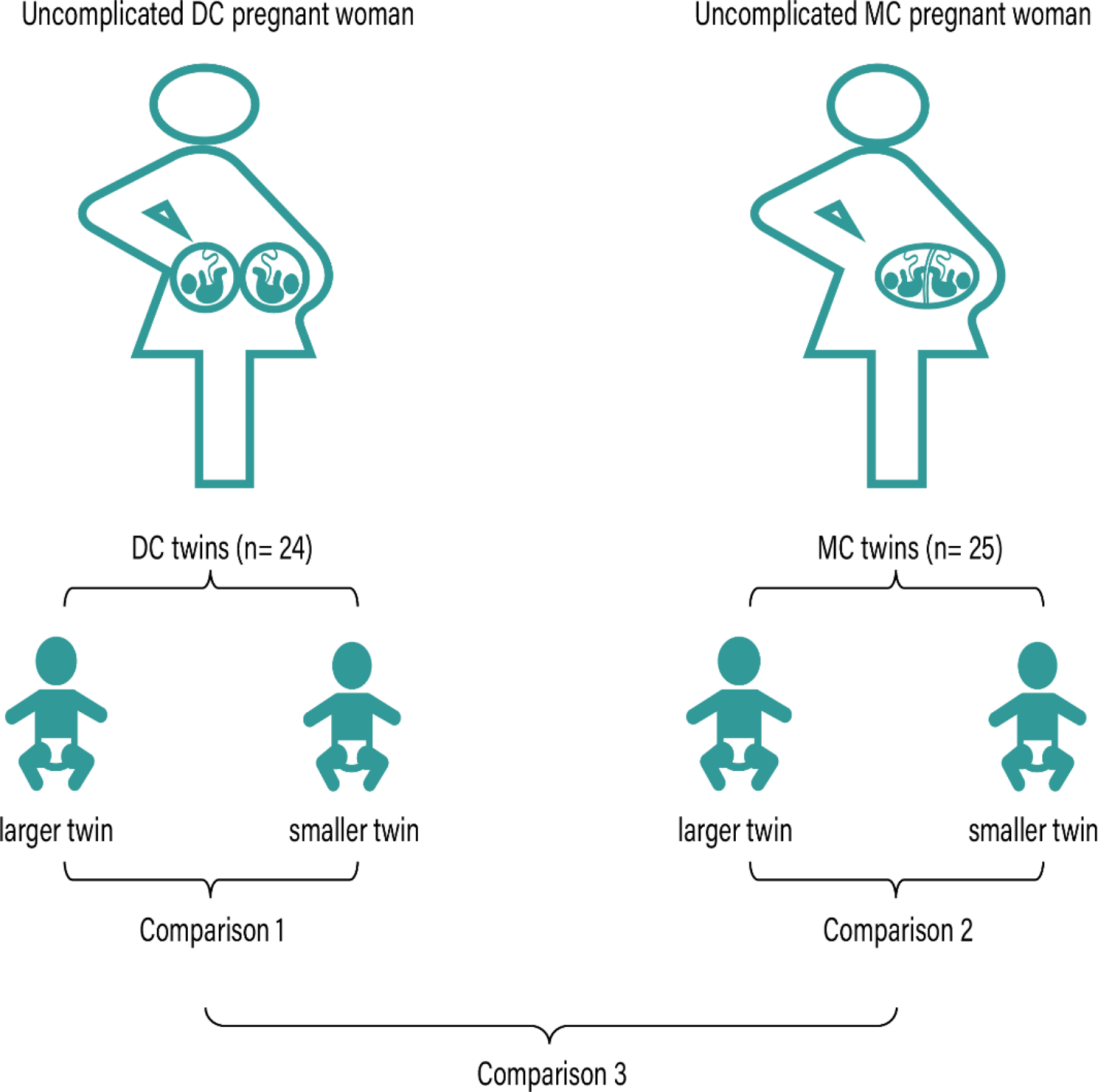
Overview of study design. 49 twin pregnancies were included in this study, including 24 DC and 25 MC pregnancies. Comparison 1 identifies within-pair metabolite variation between larger and smaller twins in DC twin pairs. Comparison 2 identified similar differences in MC twin Pairs. Comparison 3 is a comparison between DC and MC pregnancies

Women with twin pregnancies were recruited from the Peking University Third Hospital, and included in the study as part of the University Hospital Advanced Age Pregnant Cohort (clinicaltrials.gov Identifier: NCT03220750). The research protocols involving human participants were approved by The Human Research Ethics Committee of Peking University Third Hospital (IRB00006761-2016145). All participants have written informed consent prior to study recruitment. A total number of 258 twin pregnancies were enrolled in the cohort between September 2017 and December 2018. Briefly, exclusion criteria included maternal chronic diseases, malnutrition, smoking, drug abuse, fetal congenital and genetic anomalies, intrauterine fetal death (IUFD), twin-to-twin transfusion syndrome (TTTS), twin anemia polycythemia sequence (TAPS), selective intrauterine growth restriction (sIUGR), other adverse twin pregnancy outcomes, and those lost to follow-up at birth (Supplementary Fig. 1). Gestational age was determined by last menstrual period and confirmed with the ultrasound measurement of the crown-rump length (CRL) of the larger twin during 10–14 weeks’ gestation by first-trimester ultrasound examination for spontaneous conception according to the guideline of the International Society of Ultrasound in Obstetrics and Gynecology (ISUOG) [18]. After these exclusions, uncomplicated twin pregnancies with intertwin estimated fetal weight (EFW) discordances less than 25% remained in our study were 25 MC and 24 DC twin pairs. The fetal birth weight was measured by a clean electronic balance at birth. Thus, we take the following three groups for comparison: Comparison 1 compared the larger and smaller twin of DC twins (DC-L/DC-S). Comparison 2 compared the larger and smaller twin of MC twins (MC-L/MC-S). Comparison 3 compared the twins of DC and MC. All three comparisons are shown in Fig. 1.

### Ultrasound assessment

Three different obstetric sonographers were assigned to perform all ultrasound examinations using a Voluson E10 (GE Healthcare, Zipf, Austria) ultrasound machine equipped with a C5-2 convex array probe and a Rm6c volume probe on participants whose gestation age was prior to 16 weeks. After recruitment at 16 weeks of gestation, ultrasound examinations were performed by one certified twin specialist obstetric sonographer. Moreover, fetal biometry/Doppler indices of all recruited participants were reported by the same sonographer at least once every two weeks based on the recommendations of the ISUOG [19].

### Diagnosis of MC/DC pregnancy

The diagnosis of chorionicity and amnionicity was determined by a fixed obstetric sonographer. Twin pregnancy was identified based on ultrasonography conducted in the first trimester of gestation. Chorionicity was identified by (a) assessment of the membrane thickness at the insertion site of the amniotic membrane into the placenta, (b) determination of the T-sign or lambda sign between 11 and 14 gestational weeks, and (c) the number of placentas. Besides, the chorionicity of twin pregnancies was further evaluated after delivery via the pathological characteristics of the placenta.

### Sample collection

Umbilical cord blood samples were collected from each of the umbilical veins into EDTA-coated blood collection tubes immediately after delivery and processed within twelve hours. Plasma was obtained by standard density gradient centrifugation, which was centrifuged twice at 3,000 rpm for 10 min at 4 °C. And then, plasma was transferred into cryopreservation tubes (Micronic, Holland) and stored at − 80 °C until analysis.

### Metabolite extraction from cord plasma

Plasma supernatants were isolated after centrifugation at 12,000 rpm for 15 min at 4 °C. The supernatants were then dehydrated in a Speed Vac (Labconco, USA) at room temperature for 7 h. Dried supernatants were kept at − 80 °C prior to chemical derivatization.

### Methyl Chloroform Derivatization and Gas Chromatography-Mass Spectrometry (GS-MS) analysis

The extracted samples were chemically derivatized via the methyl chloroformate (MCF) method based on the recommendations published by Smart et al. [20]. All cord plasma samples were analyzed in a single batch and derivatized compounds were examined by an Agilent GC7890B system using a ZB-1701 GC capillary column. An MSD5977A mass selective detector with the electron impact voltage set to 70 eV was applied to analyze the compounds. The GC column used for metabolite separation was the ZB-1701 GC capillary column (30 m × 250 μm id × 0.15 μm with a 5 m guard column, Phenomenex). The GC temperature was set up according to the protocol of Han et al. [21].

### Metabolite identification, GS-MS data mining, and data normalization

The automated Mass Spectral Deconvolution and Identification System (AMDIS) was implemented for deconvolution. Metabolite identifications were determined based on the MS fragmentation pattern and respective chromatographic retention time via the in-house MCF mass spectral library established by Silas Villas Boas’s metabolomics laboratory in New Zealand. The peak height of the most abundant fragmented ion mass was selected using the MassOmics R scripts to extract the relative concentrations of the identified metabolites. The identified compound’s abundances were then normalized by the relative level of the internal standard (D4-alanine, D5-phenylalanine, or D2-tyrosine) in the corresponding sample. Median centering was performed to remove batch variation via nine QC samples (three QC samples per batch), and the dilution effects of plasma were corrected by a total ion chromatogram. Then, blank samples were used to subtract background contamination and carryover effects from identified metabolites.

### Statistical analysis

Prior to statistical analysis, the cord plasma metabolite levels were adjusted by log transformation and Pareto scaling in order to provide the best Gaussian distribution for the dataset. Student’s t-test, non-parametric Mann-Whitney U test, Chi-square test, and Fisher’s exact test were performed in R to investigate prenatal clinical characteristics. Partial least squares discriminant analysis (PLS-DA) was performed using the MetaboAnalyst 5.0 package for R to screen for significant metabolites and identify metabolic profile differences between twin groups (http://www.metaboanalyst.ca). The linear logistic regression model was implicated in detecting differential metabolites between comparisons without the influence of confounding factor (e.g. gestation age and the way of conception) using the general linear model (glm) logistic regression package in R. Generalized estimating equation (GEE) modeling was utilized to examine metabolite correlations with birthweight discordance both within and between twin pairs. Metabolite pathway activity was calculated based on the KEGG database using Metaboanalyst 5.0. Metabolic networks of interest were reconstructed using the metascape package in Cytoscape (Version 3.9.1). Heatmaps, line graphs, and circus plots were made using the ggplot2 and GOplot R-packages [22, 23]. The intra- and inter-observer reliabilities of the CRL ultrasound measurement were determined using the Bland-Altman method [24] using Irr R package.

## Results

### Population characteristics

Characteristics of women pregnant with MC or DC twins included in this study are listed in Table 1. The postnatal outcomes of MC twins and DC twins are summarized in Table 2. No differences were observed between groups regarding pre-gestational maternal body mass index, weight gain during pregnancy, mode of delivery, maternal age, neonatal sex, Apgar score (1 and 5 min), and birth weight discrepancy with co-twins (comparison 1 and 2). In contrast, the gestational age at delivery, average birthweight, and mode of conception were significantly different between MC and DC groups (comparison 3). This was in part due to the fact that MC pregnancies were generally delivered earlier to avoid the higher risk of complications than DC pregnancies. In vitro fertilization (IVF) dominated the mode of conception in our DC pregnancies (79.17%, p < 0.001). Indicators of fetal development, including fetal abdominal circumference (p < 0.01), height (p < 0.05), fetal head circumference (p < 0.05), and birthweight (p < 0.01), were different between the larger twin and the smaller twin of DC groups.

**Table 1:** Comparison of the clinical characteristics of MC and DC groups

Maternal Characteristics	MC Group(n = 25)	DC Group(n = 24)	P-Value
**Maternal age (years)**	31(3)	33(4)	0.17^a^
**Pre-gestational body mass index (kg/m** ^**2**^ **)**	21.7(19.5,25.3)	20.65(19.1,22.3)	0.23^b^
**Weight gain during pregnancy (kg)**	16.02(3.66)	18.04(4.43)	0.09^a^
**Gestational age at delivery (wks)**	36(35,37)	37(37,37)	0.02^b*^
**Delivery**			1^d^
**Cesarean**	24(96%)	24(100%)	
**Vaginal**	1(4%)	0(0%)	
**IVF-ET/Natural conception**			0.00^c**^
IVF-ET	5(20%)	19(79.17%)	
Natural conception	20(80%)	5(20.83%)	

^a^ Student’s T-test. ^b^ Mann-Whitney U test. ^C^ Chi-square test. ^d^ Fisher’s exact test, ^e^ IVF-ET, In Vitro Fertilization & Embryo Transfer, * P-value < 0.05. ** P-value < 0.001

**Table 2:** Comparison of postnatal outcomes in the MC and DC twins

Postnatal outcomes	MC Group			DC Group		
	TwinL(n = 25)	TwinS(n = 25)	P-Value	TwinL(n = 24)	TwinS(n = 24)	P-Value
**Birth weight(g)**	2492.8 ± 353	2308.4 ± 344	0.068^a^	2781.67 ± 292	2551.25 ± 286	0.0082^a*^
**Apgar score at 1 min**	10(10,10)	10(10,10)	0.52^b^	10(10,10)	10(10,10)	1^b^
**Apgar score at 5 min**	10(10,10)	10(10,10)	0.57^b^	10(10,10)	10(10,10)	1^b^
**AC**	31(30,33)	30(30,32)	0.052^b^	32(31,33)	31(30,32)	0.011^b*^
**HC**	33(32,34)	33(32,33)	0.052^b^	33.5(33,34.25)	33(32,34)	0.027^b*^
**Height**	46(45,47)	46(44,47)	0.29^b^	47(46.75,48)	46(45,47)	0.015^b*^
**Birth weight discrepancy of twin(g)**	170(110,280)			225(87.5,312.5)		0.40^b^
**Average birth weight(g)**	2480(2180,2695)			2697.5(2468.75,2817.5)		0.00022^b**^
**Neonatal sex**						0.56^c^
Male	32(64%)			27(56.25%)		
Female	18(36%)			21(43.75%)		

^a^ Student’s T-test. ^b^ Mann-Whitney U test.^c^ Chi-square test. AC, Abdominal circumference. HC, head circumference. L = larger, S = smaller, * P-value < 0.05, ** P-value < 0.001

### Analysis of the umbilical cord plasma metabolome profiles in MC and DC twins

**Fig. 2:**
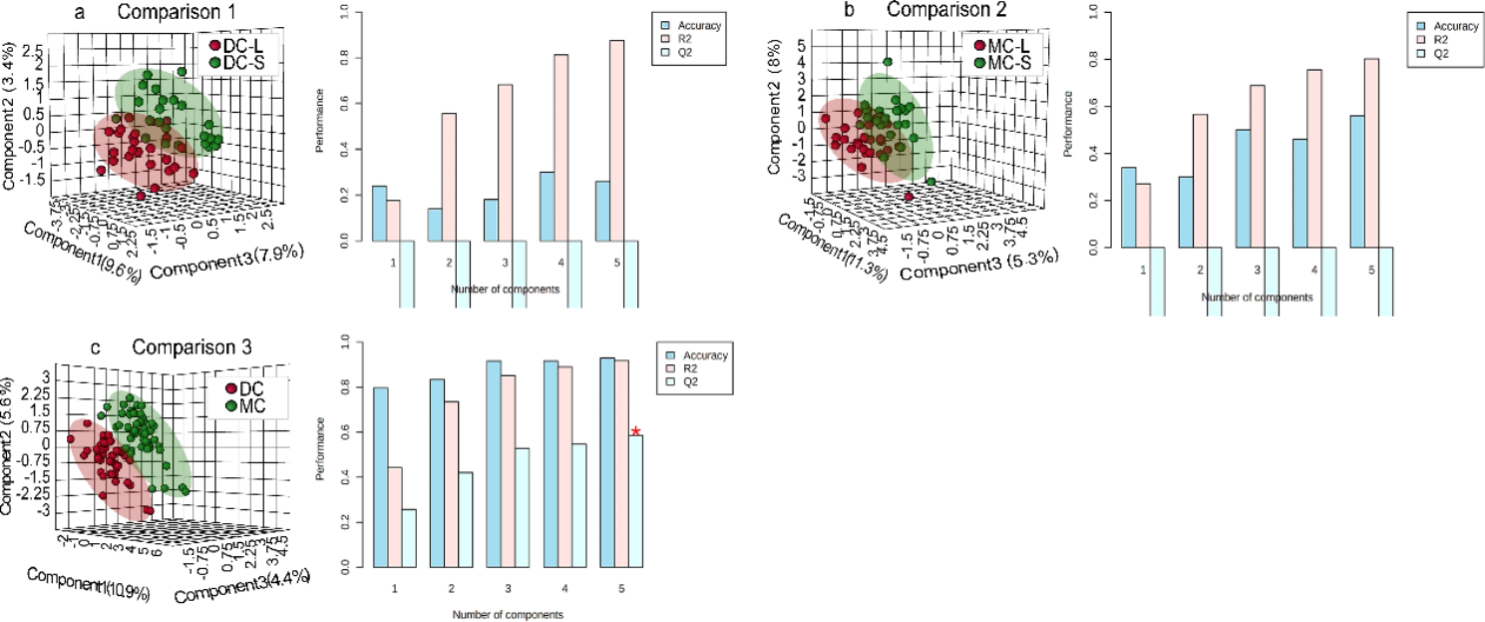
Partial least squares discriminant analysis (PLS-DA) of the umbilical cord plasma metabolome between the three twin comparisons, including a measure of prediction model performance (right bar graphs). The right bar graphics are leave-one-out cross-validations (LOOCV), where R2 indicates how well the model explains the data and Q2 indicates the reproducibility of the PLS-DA model. The red asterisk indicates the best classifier. List of abbreviations; MC = Monochorionic; DC = Dichorionic; L = Larger twin; S = Smaller twin

**Fig. 3:**
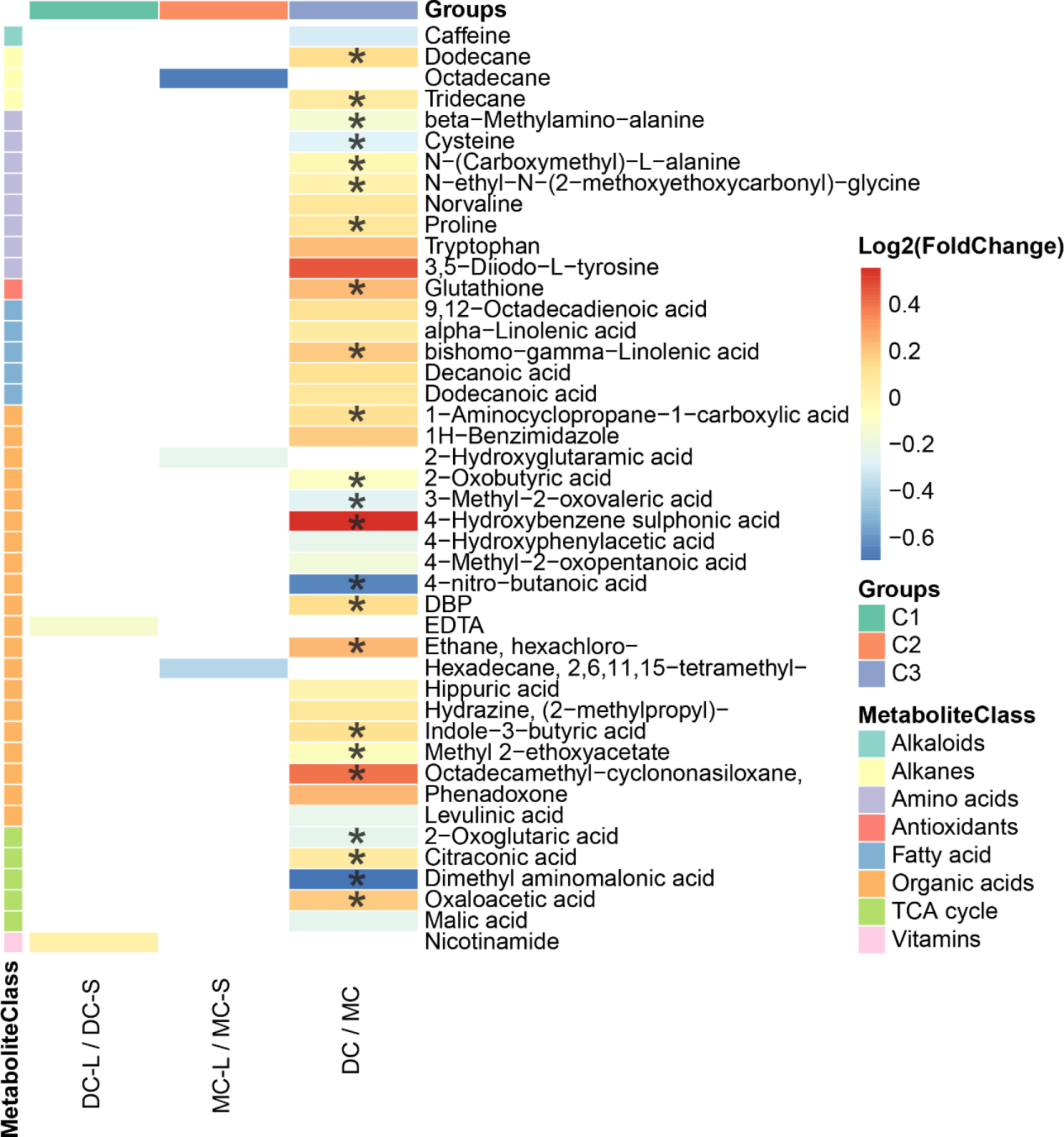
The heatmap illustrates the differences in the umbilical cord plasma metabolome between the three groups of DC twins and MC twins. The relative concentrations of umbilical cord plasma metabolites are shown via a log2(foldchange). Red color blocks represent higher metabolites levels in dividend groups than the divisor groups, whereas blue color blocks represent lower metabolites levels in dividend groups than the divisor groups. Only the metabolites with a p-value less than 0.05 (The logistic regression adjusted for gestational age and the way of conception) were displayed. The metabolites with a significant p-value less than 0.05 and a q-value less than 0.05 were marked via a black asterisk. List of abbreviations: DC = Dichorionic; MC = Monochorionic; L = Larger twin; S = Smaller twin

The metabolites of twin umbilical cord plasma samples were identified using our in-house MCF mass spectral library and NIST library (https://www.nist.gov/nist-research-library) with the inter-assay coefficient of variation in QC samples ranging from 0.9 to 23.0% (See Supplementary Table 1). Representative metabolites are indicated on a GC-chromatogram displayed in Supplementary Fig. 2. Umbilical cord plasma of DC co-twins demonstrated better global metabolomic separation of larger and smaller twins compared to the MC co-twins (Fig. 2a and b). Lower plasma levels of EDTA and higher plasma levels of nicotinamide discriminated larger from smaller twins within DC pairs with a p-value less than 0.05 (C1, Fig. 3), while octadecane, hexadecane,2,6,11,15-tetramethyl, and 2-hydroxyglutaramic acid was significantly higher in smaller MC twins (p < 0.05) relative to their larger co-twin (C2, Fig. 3). PLS-DA score plots (Fig. 2) illustrate that the metabolic profiles of MC twins as a group were generally different from DC twins (Fig. 2C). The results of comparison 3 (C3) between DC and MC displayed a valid model performance (Accuracy = 0.93, R2 = 0.92, Q2 = 0.59); the major three latent variables accounted for 10.9%, 5.6%, and 4.4% of the variation in the metabolite levels. The most distinct separations and the most valid LOOCV were demonstrated when comparing DC and MC (Fig. 2, C). Adjusted logistic regression was performed for all comparisons to account for the potential influencing factors of gestational age at delivery and mode of conception. This revealed 23 significant plasma metabolites that contributed to the separation of the MC twins and DC twins with a p-value and q-value less than 0.05 and 0.05 respectively (C3; Fig. 3). Among them, two amino acids and one saturated fatty acid were found at higher levels in DC twins.

### Within and between pair correlation of metabolites with birthweight discordance and birthweight

**Fig. 4:**
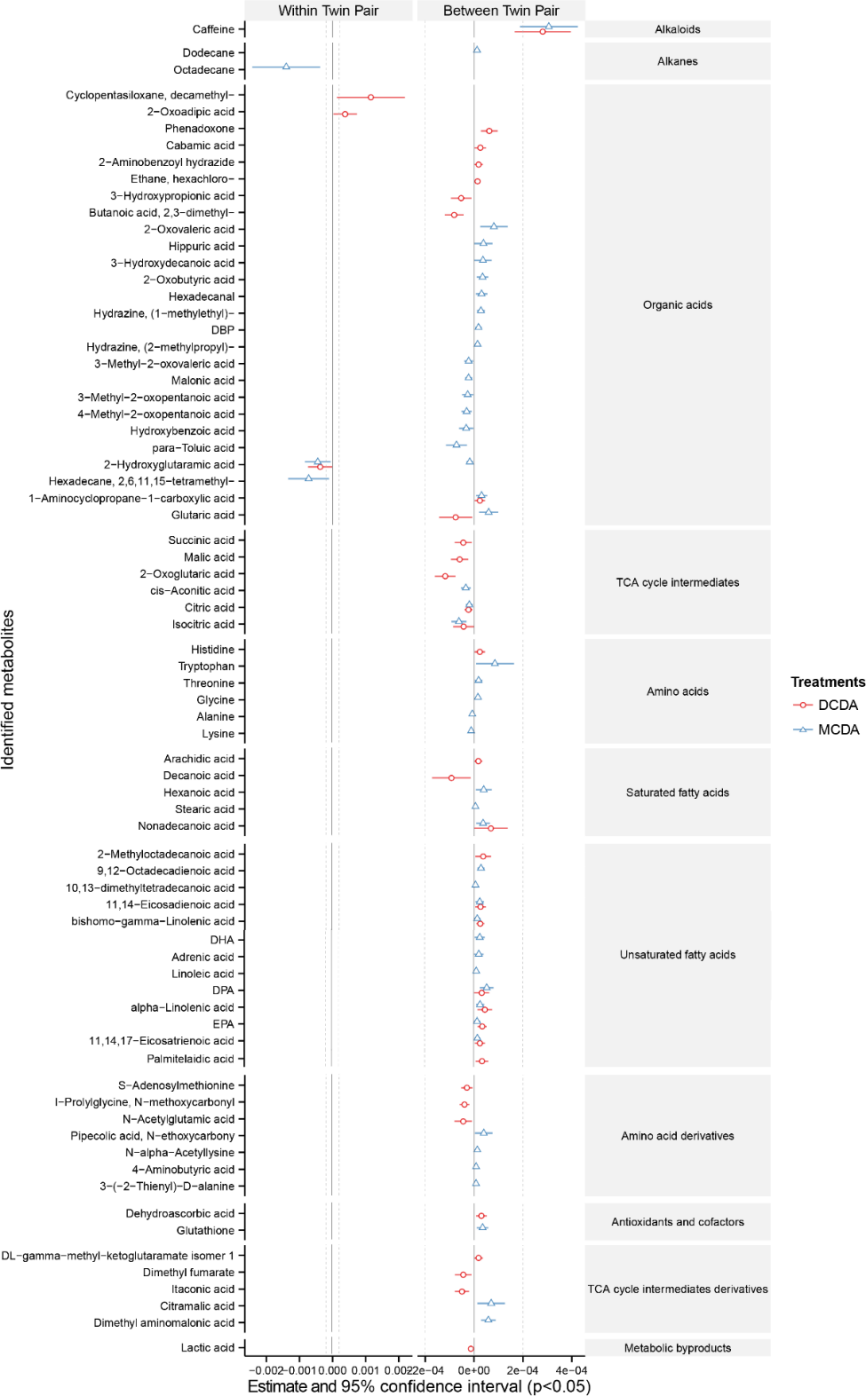
Correlation of birth weight within (weight discordance within larger and smaller co-twin, left column) and between (average birth weight between twin pairs, right column) twin pairs of umbilical cord plasma metabolites detected from DC and MC twins, analyzed using a generalized estimating equation (GEE). The red lines represent the 95% confidence intervals for the correlation of metabolites with weight discordance in the DC umbilical cord plasma. The blue lines represent the 95% confidence intervals for correlating metabolites with weight discordance in MC umbilical cord plasma. The center dotted line in each column indicates 0 correlation; metabolites to the right of the dotted line are positively correlated with weight discordance, whereas metabolites to the left of the dotted line are negatively correlated with weight discordance. In addition, the distance between the metabolites and the dotted line represents the strength of the correlation. Metabolites are classified in accordance with their chemical properties, and only the metabolites significantly correlated with birth weight (p-value < 0.05) are plotted

A GEE regression model was applied to measure the level of correlation between plasma metabolites and birth weight discordance within pairs (smaller vs. larger twin) and between twin pairs, accounting for the individual (twin) and shared (maternal) factors (Fig. 4). Octadecane and 2,6,11,15-tetramethyl-hexadecane were negatively associated with birthweight discordance within MC twin pairs only, while decamethyl-cyclopentasiloxane and 2-oxoadipic acid were positively associated with birthweight discrepancy in DC twins only. 2 − hydroxyglutaramic acid levels were negatively associated with birthweight within both MC and DC twin pairs. A total of 70 metabolites were found to be associated with the birthweight following twin pair comparisons. This included unsaturated fatty acids, TCA cycle intermediates, antioxidants, as well as most amino acids, amino acids derivatives, TCA cycle intermediates derivatives, and organic acids (Fig. 4).

### Pathway enrichment analysis of MC twins and DC twins

**Fig. 5:**
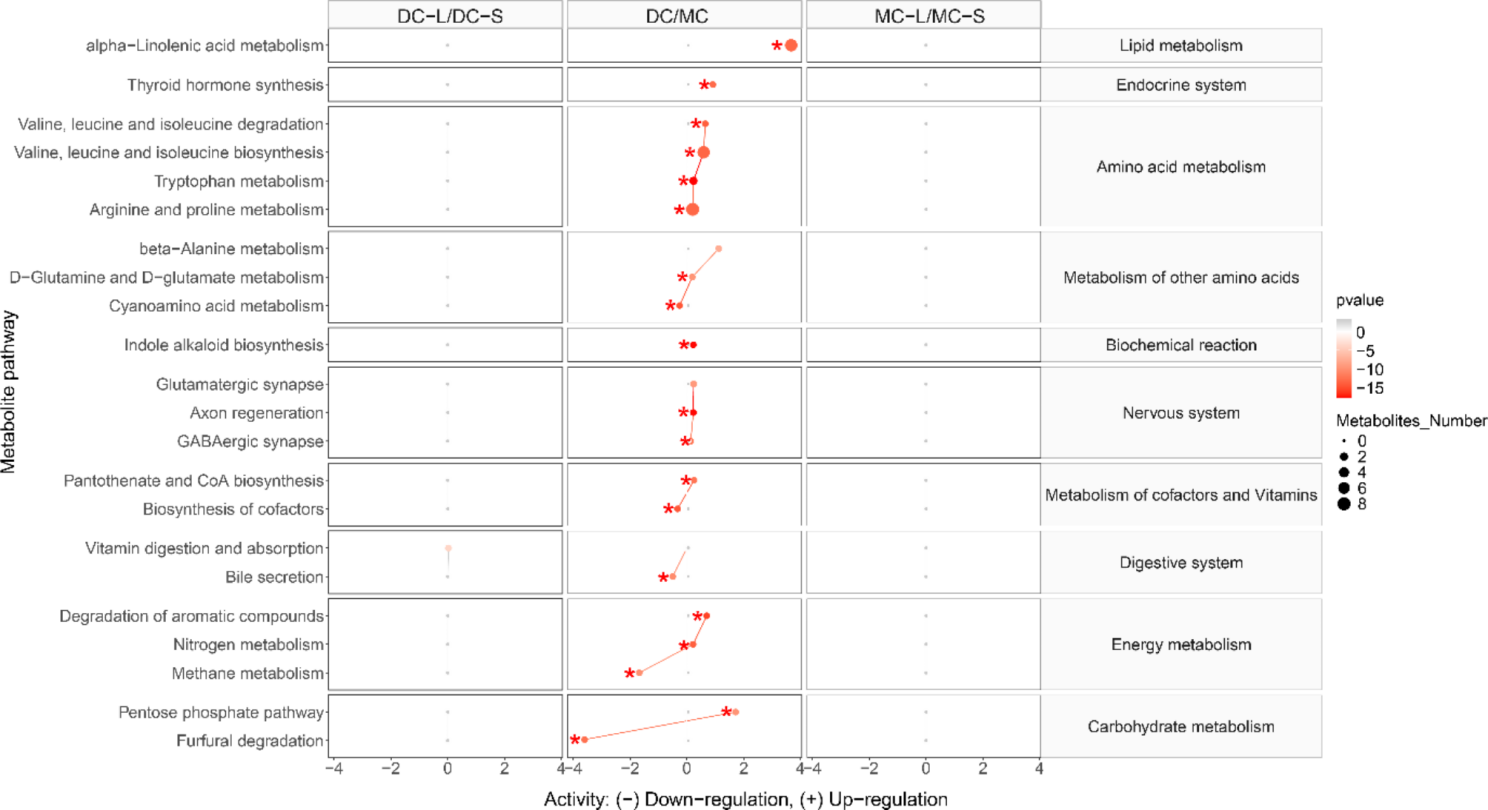
The predicted metabolic activity in the umbilical cord plasma associated with DC twins and MC twins was illustrated using log_2_(fold change). The black dotted line in each column indicates metabolic pathways in the divisor groups that were adjusted to 0. The red plots at positive values represent upregulated metabolic activity in dividend groups compared to the divisor groups, whereas the red plots at negative values represent downregulated metabolic activity in dividend groups compared to the divisor groups. The red dot sizes represent the enrichment ratio of pathway computed by metabolite hits. Only the metabolic pathways with a significant p-value less than 0.05 (Logistic regression) and a q-value less than 0.1 (false discovery rate) are plotted. The pathways with a significant p-value less than 0.05 and a q-value less than 0.05 were marked via red asterisks. List of abbreviations: DC = Dichorionic; MC = Monochorionic; L = Larger twin; S = Smaller twin

**Fig. 6:**
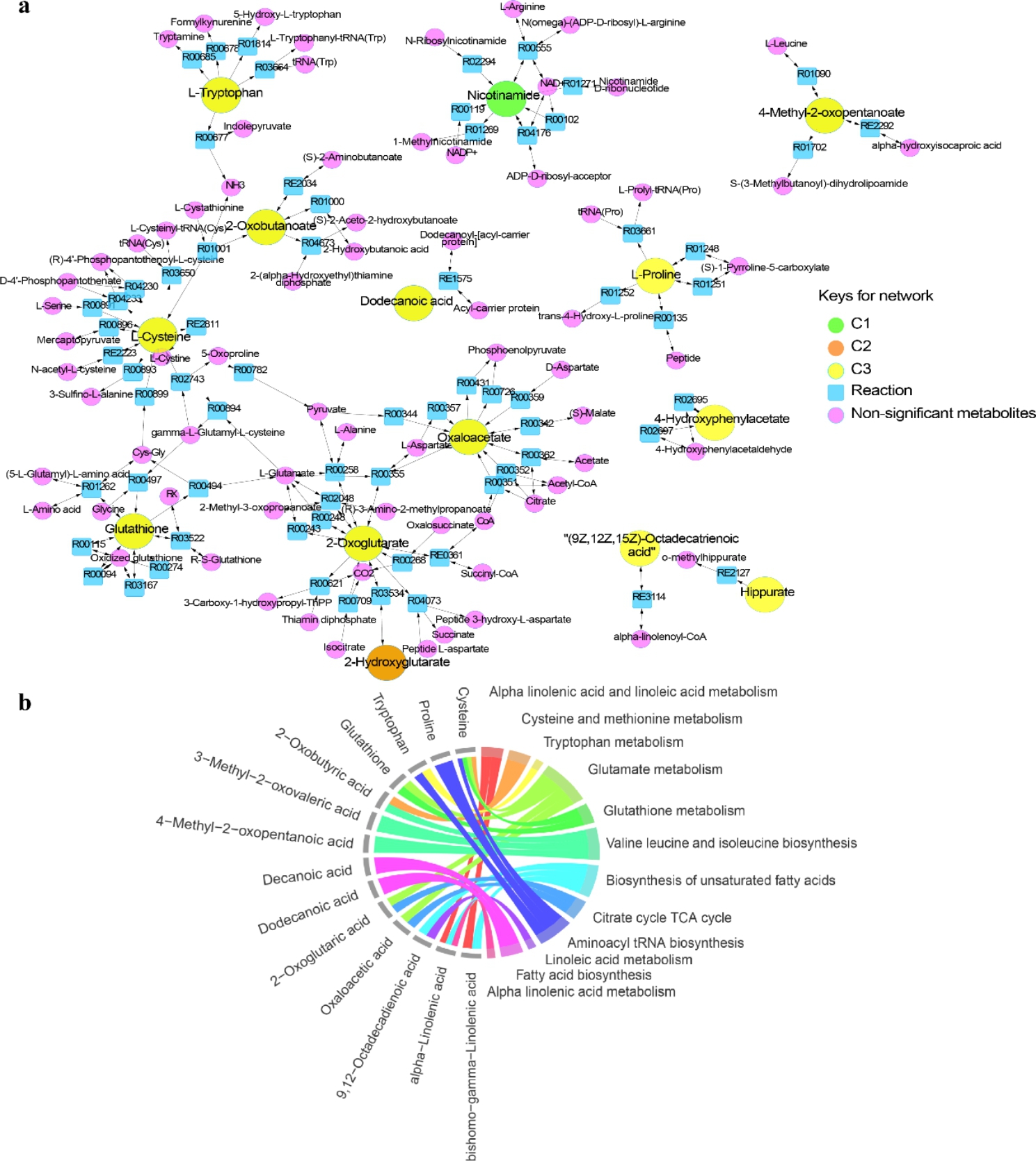
The metabolic networks represented in the umbilical cord plasma metabolome of uncomplicated MC twins. (**a**) The two-dimensional network was constructed using the metabolic pathways that were significantly different between the MC twins and the DC twins. The green circles are significantly different metabolites between the DC-L and DC-S abbreviated as C1. The orange circles are metabolites that were significantly different between the MC-L and MC-S abbreviated as C2. The yellow circles are metabolites that were significantly different between the DC and MC abbreviated as C3. The blue squares are the metabolic reactions associated with significant metabolites. All purple circles are unidentified metabolites that were directly linked to identified metabolites. The arrowheads indicate the direction of the metabolic reactions. (**b**) A chord plot displays how metabolites with p < 0.05 participate in different significant metabolic pathways

The differences in metabolic pathway activities derived from the identified metabolites in umbilical cord plasma samples appear to be associated with chorionicity. The majority of metabolic pathways we observed were upregulated in comparison 3 (DC/MC) (Fig. 5). Lipid metabolism, one metabolism of the endocrine system, four pathways associated with amino acids metabolism, three metabolisms of the nervous system, and two energy metabolisms were found in lower levels in the MC compared to the DC twins. However, no significant metabolic pathway differences were detected in comparison 2 (MC-L/MC-S). Vitamin digestion and absorption were upregulated in larger DC twins (p = 0.069). The significant pathways were linked to their shared metabolites and reconstructed into a metabolic network and a chord plot based on the KEGG metabolic framework via Cytoscape (Fig. 6). Eight metabolites were significantly different between DC and MC twins including tryptophan, 2-oxobutanoate, cysteine, glutathione, dodecanoic acid, oxaloacetate, 2-oxoglutarate, and 4-methyl-2-oxopentanoate (Fig. 6a). Four significant metabolites were involved in the glutamate metabolism including cysteine, glutathione, oxaloacetate, and 2-oxoglutarate (Fig. 6b).

### Inter- and intra-observer variations for ultrasound measurements

The determination of gestational age using CRL ultrasound indicated that inter-observer reliability agreement between observers 1 and 2 was 0.994 (95% CI 0.986–0.998), whilst the intra-observer agreement of CRL measurement was 0.997 (95% CI 0.992–0.999) and 0.996 (95% CI 0.989–0.998) for observer 1 and 2 correspondingly (**Supplementary Fig. 3**). These outcomes indicated good reproducibility of the CRL ultrasound measurement to determine gestational age in our hospital.

## Discussion

In this study, we aimed to identify metabolomic differences in newborn twins associated with chorionicity (MC vs. DC) and to relate these to birthweight and birthweight discordance. The different metabolic profiles identified highlight the difference in the intrauterine growth environment experienced by twins in association with the mode of placentation. We observed more similarities in infant outcomes and metabolite profiles in MC co-twin pairs compared to DC co-twin pairs. Higher concentrations of most amino acids and organic acids were revealed in DC twins compared to MC twins associated with chorionicity. The accumulations of most nutritional metabolites and exogenous substances were positively correlated with the birthweight of both DC and MC twins. In contrast, 2 − hydroxyglutaramic acid (2-HG) was negatively correlated with birthweight discordance of both DC and MC twins.

**Fig. 7:**
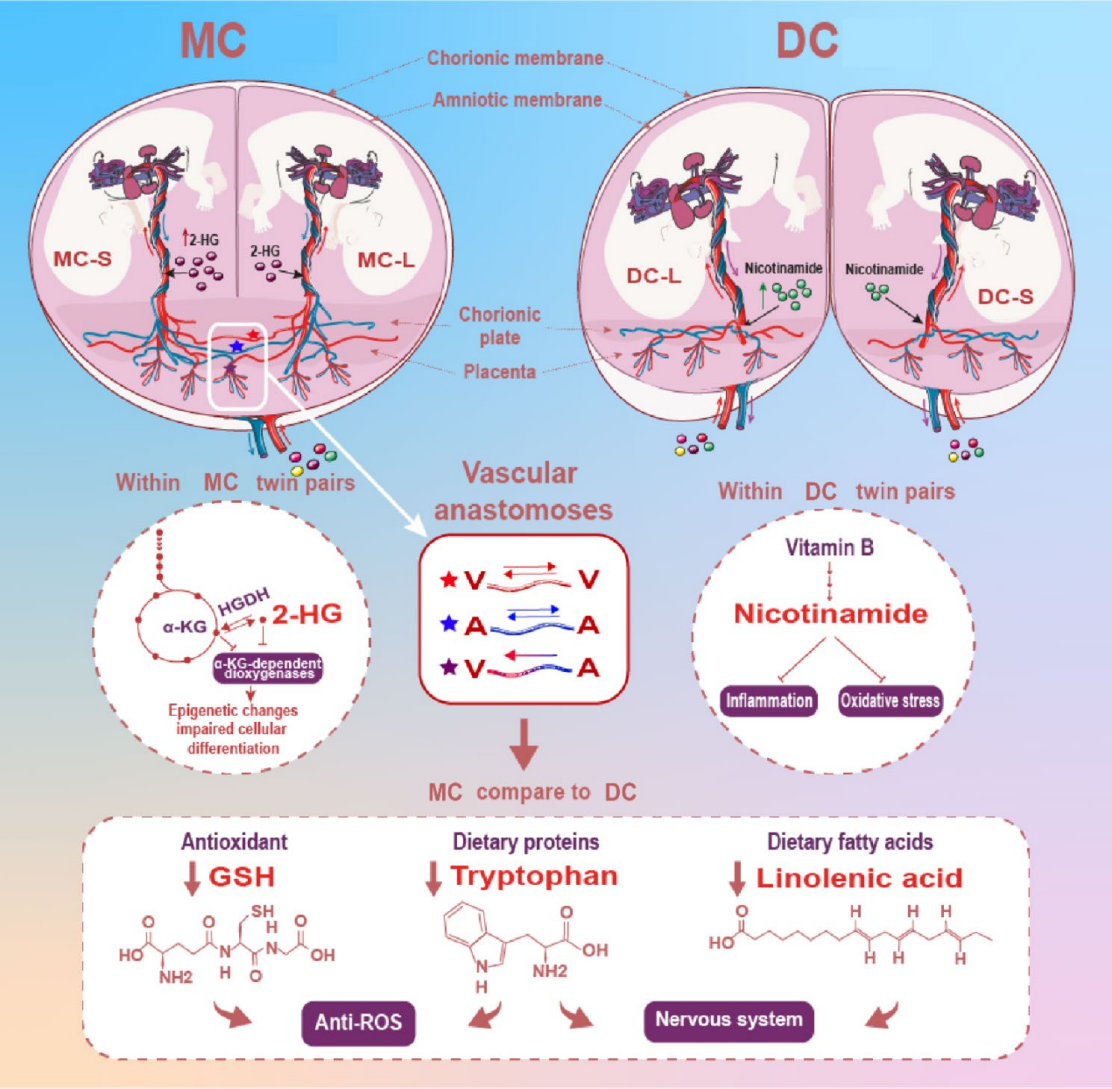
Changes in the metabolic phenotypes of twin cord blood between differential chorionicities. Uncomplicated monochorionic twin pregnancy shows three types of anastomoses: the veno-venous (VV) anastomosis is bi-directional occurred between the red veins (red star), large arterio-arterial (AA) anastomosis is bi-directional occurred between the blue arteries (blue star), and arterio-venous (AV) anastomosis is unidirectional from blue arteries to red veins (purple star). The umbilical cord plasma discrepancies within twin pairs and between twin pairs were exhibited. Within twin pairs: a higher 2-HG level was observed in the MC smaller twin compared to the larger one; a higher nicotinamide level was observed in the DC larger twin compared to the smaller one. Between MC and DC twins: the GSH metabolism, tryptophan metabolism, and linolenic acid metabolism were downregulated in the MC twins. List of abbreviations: DC = Dichorionic; MC = Monochorionic; L = Larger twin; S = Smaller twin; 2-HG, 2 − hydroxyglutaramic acid; α-KG, α-Ketoglutaric acid; HGDH, 2-Hydroxyglutarate dehydrogenase; GSH, glutathione

The most interesting findings to emerge from the within-twin pair’s analysis were that higher plasma concentrations of 2-HG only tended to be associated with the metabolite differences identified in MC co-twins. In contrast, the metabolite nicotinamide contributed to differ in DC twin pairs only. GSH metabolism, tryptophan metabolism, and fatty acid metabolism were significantly downregulated in MC twins relative to DC twins in the between-pair analyses (Fig. 7). These metabolic alterations in the umbilical cord blood appear to serve as an essential reflection of the unequal blood distribution resulting in differentiation of chorionicity, thus influencing twins’ growth.

### Differential metabolites within MC twin pairs

Growth in monochorionic twins is determined by the same genetic potential, as well as by the placental sharing and vascular anastomoses [25–27]. The present study demonstrated that less birthweight discrepancy was observed in uncomplicated MC twin pairs compared to DC twin pairs. After eliminating the potential influence of gestation age and IVF-ET, we found that the spatial images generated by PLS-DA displayed more similar metabolite profiles within MC twins than in DC twins (Fig. 2). Indeed, placental vascular anastomoses that connect the two circulations of monochorionic twin pregnancies account for the equalization of oxygen and nutritional supply between larger and smaller twins [25, 26]. Surprisingly, higher cord blood concentrations of 2-HG, octadecane, and 2,6,11,15-tetramethyl hexadecane were found in MC smaller babies than in their larger siblings (Fig. 3). A negative correlation between 2-HG levels and twin birth weight was also observed in this study (Fig. 4). Literature reported that a higher level of 2-HG has been identified as a result of abnormal metabolism under homeostasis [28]. The accumulation of 2-HG acted as a competitive inhibitor of alpha-ketoglutarate-dependent dioxygenases, causing profound metabolic and epigenetic dysregulation [29, 30]. A recent study showed that preventing the reduction of 2-HG levels after fertilization would impede the erasure of histone modifications such as H3K4me3 [31]. This highlights the potential role associated with epigenetic network remodelling of 2-HG in fetal development.

### Differential metabolites within DC twin pairs

The second interesting observation of the current study is that nicotinamide was upregulated in DC larger compared to smaller DC twins, while no such disparity was observed within MC twin pairs (Fig. 3). Nicotinamide is an amide form of vitamin B3 as an indispensable nutrient often supplemented to pregnant women for embryonic development. It has been reported that nicotinamide exerts anti-inflammatory and antioxidative properties in the human placenta [32]. Inflammation plays a pivotal role during human pregnancy. F. Li et al. [33] demonstrated that dietary nicotinamide benefits both dams and pups with preeclampsia characterized by an excessive inflammatory response. A cross-sectional study design that included 626 mothers and their singleton offspring also reported that maternal vitamin B3 intake in pregnancy contributes to increasing birthweight [34]. It will be interesting to validate that adequate niacinamide supplementation may benefit the birthweight of twins.

### The metabolic disparities between MC and DC twins related to vascular anastomoses

A hypothesis proposed by Sebire et al. [35] is that anastomoses between placental circulations in monochorionic twins develop randomly during early pregnancy. Subsequently, placental growth is influenced by spontaneous closure or disruption of anastomoses. Asymmetrical loss of anastomoses may trigger TTTS due to disturbed hemodynamics [35]. An imbalance of angiogenesis is more vulnerable to oxidative stress, which could increase the likelihood of intrauterine growth restriction, fetal death, spontaneous preterm labour, and preeclampsia. The findings from our umbilical plasma metabolome analysis illustrated that antioxidants (glutathione, GSH) were downregulated in MC twin pairs (Fig. 3). GSH is an intracellular tripeptide composed of glutamate, glycine, and cysteine, which protects the organism from oxidative stress by eliminating reactive oxygen species (ROS). Altered cysteine and glycine derivatives (Glycine, N-ethyl-N-(2-methoxyethoxycarbonyl)-, 2-methoxyethyl) were discovered in comparison 3, as illustrated in Fig. 3. The alterations of other essential metabolites involved in glutamate metabolism were also observed, including 2-oxoglutaric acid and oxaloacetic acid (Fig. 6b). In addition, our reconstructed metabolic network indicated that glutathione interacted with cysteine during pregnancy (Fig. 6a). Several studies supported that the regulation of antioxidant levels and their metabolites may be related to the maintenance of the redox state and blood vessel formation [17, 36, 37]. These findings support that the disproportionate vascular anastomoses in the MC twins are associated with oxidative stress, thus affecting the regulation of the antioxidants and their metabolites.

Interstitial vascular anastomose in twin pregnancy has also been known as a strong factor influencing the blood distribution and results in disturbed nutrient supply. A prospective observational study provided evidence that underlying hemodynamic changes also occur in uncomplicated MC twins [38]. Previous studies reported that the higher rates of abnormal placental vascular observed in MC twins compared to DC twins, even in the uncomplicated MC twin pregnancies, were associated with neonatal encephalopathy and cerebral palsy [39–41]. We observed that tryptophan metabolism was downregulated in MC twins (Fig. 5). Tryptophan is an essential amino acid, which must be obtained exclusively from a dietary source in humans. Disrupted tryptophan metabolism was associated with the decrease of neuronal protection for fetal growth and immunosuppressive against fetal rejection [42]. Consistent with the literature, this research found that the metabolic pathways related to the nervous system were impaired in MC twins (Fig. 5). Furthermore, we observed that three unsaturated fatty acids and two saturated fatty acids were downregulated in the MC twins (Fig. 3). Among them, linolenic acid and its derivatives were positively associated with the birthweight of twins (Fig. 4). It has been reported that prenatal administration of linolenic acid benefits neurogenesis and the cognitive abilities of mice with Down syndrome [43]. These results demonstrated that the placental vascular anastomoses in MC twins were related to the imbalanced nutrient distribution, thereby affecting their downstream metabolic pathway such as nervous system metabolism.

### The accumulations of exogenous substances in MC and DC twins

Lastly, we found that a higher caffeine level was discovered in MC twins and the enrichment of caffeine in umbilical cord plasma was positively correlated with birth weight both in MC and DC twins (Figs. 3 and 4). Several studies suggested that caffeine was consistently elevated during pregnancy, and the elevation might be due to a slower caffeine metabolism in pregnant women rather than an increase in coffee intake [44, 45]. Together, the caffeine concentration of twins’ cord blood increases with gestational age and positively correlates with progressive fetal weight. Secondly, the xenobiotic compound decamethyl-cyclopentasiloxane (D5) was positively correlated with birth weight discordance between DC co-twins (Fig. 4). It is one of the cyclic siloxanes widely used in cosmetics and body care products. Although limited research has reported that the other cyclic siloxanes may impair the fertility and reproduction of female rats [46, 47]. The role of D5 in fetal development remains largely unknown. Therefore, our study suggested that the twins may suffer from a slower metabolism of some exogenous substances, like caffeine and D5. The mechanistic function of these accumulated metabolites in twins’ growth was clearly worth revealing.

Lastly, this study has presented several limitations. The metabolic profile of umbilical blood was collected at delivery, which is metabolically different from that at early or middle gestation period. Another concern was that the sample size was small (low power for cause-effect relationship) due to the high rate of complications in twin pregnancies. In the future, the sample size and different biospecimens should be expanded. Maternal blood composition, placental pathophysiological structure, and fetal metabolism should be further investigated to better understand the influence of chorionicity on maternal-fetal transfer and the growth discordance of twins.

## Conclusions

To our knowledge, the present study is the first to investigate the metabolic profile of the umbilical cord plasma between uncomplicated MC and DC twin pregnancies. The main findings fill the gap in the scientific literature on the association between the metabolites in cord plasma and the development of uncomplicated twins. The discrepancy of umbilical cord plasma metabolome within and between MC and DC twins seems to have resulted from the alterations of metabolite allocation, such as 2-HG, nicotinamide, GSH, tryptophan, linolenic acid, and is related to discordant birthweight. The underlying mechanisms of vascular anastomosis and accumulated exogenous substances in the development of twins were still worthy of further investigation.

## Electronic supplementary material

Below is the link to the electronic supplementary material.

**Supplementary Material 1 Table 1:** Identified metabolites

**Supplementary Material 2 Fig. 1:** Flowchart of the selection of study participant

**Supplementary Material 3 Fig. 2:** Representative total ion chromatogram (TIC) of the umbilical cord plasma metabolome

**Supplementary Material 4 Fig. 3:** Intra-observer 1 variability (a), intra-observer 2 variability (b), and inter-observer variability (c) for determining gestational age. The proportional difference from the average for the crown?rump lengths (CRL) of 20 larger twins were diagnosed by two independent registered sonographers (observer 1 and 2). The upper and lower red dot lines mean the 2.5th and 97.5th percentiles for limits of agreement. The middle black line is the mean

## Data Availability

The datasets used and/or analysed during the current study are available from the corresponding author on reasonable request.

## List of abbreviations

AC: Abdominal circumference
AMDIS: Automated Mass Spectral Deconvolution and Identification System
AUC: Area Under Curve
CRL: Crown rump length
DC: Dichorionic
EFW: Estimated fetal weight
FDR: False discovery rate
GC-MS: Gas chromatography-mass spectrometry
GEE: Generalized estimating equation.
GLM: General linear model
GSH: Glutathione
HC: Head circumference
HG: Hydroxyglutaramic acid
ISUOG: International Society of Ultrasound in Obstetrics and Gynecology
IUFD: Intrauterine fetal death
IVF-ET: In Vitro Fertilization & Embryo Transfer
KEGG: Kyoto Encyclopedia of Genes and Genomes
LOOCV: Leave-one-out cross validation
MC: Monochorionic
MCF: Methyl chloroformate
PLS-DA: Partial least squares discriminant analysis
QC: Quality control
ROC: Receiver operating characteristic
ROS: Reactive oxygen species
sIUGR: Selective intrauterine growth restriction
TAPS: Twin anemia polycythemia sequence
TTTS: Twin-to-twin transfusion syndrome
