# Supplementary figures and images for: Chorionicity-associated variation in metabolic phenotype of cord blood in twin
Source: Nutr Metab (Lond). 2023 Jul 13;20:31. doi: 10.1186/s12986-023-00744-1 (PMC10339575; doi:10.1186/s12986-023-00744-1)

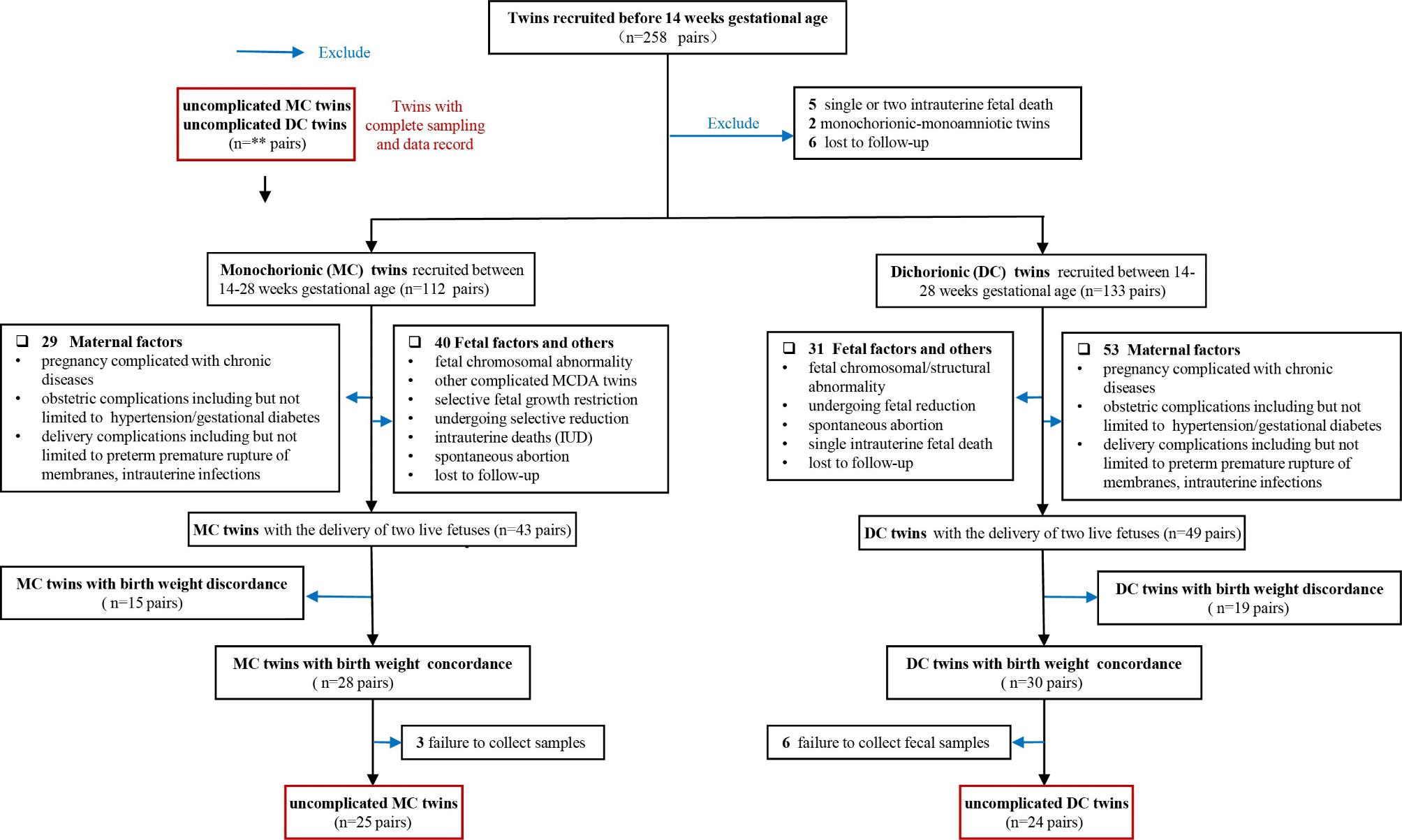

Supplement: Supplementary file 2 — Supplementary Material 2 Fig. 1: Flowchart of the selection of study participant [file 12986_2023_744_MOESM2_ESM.png]

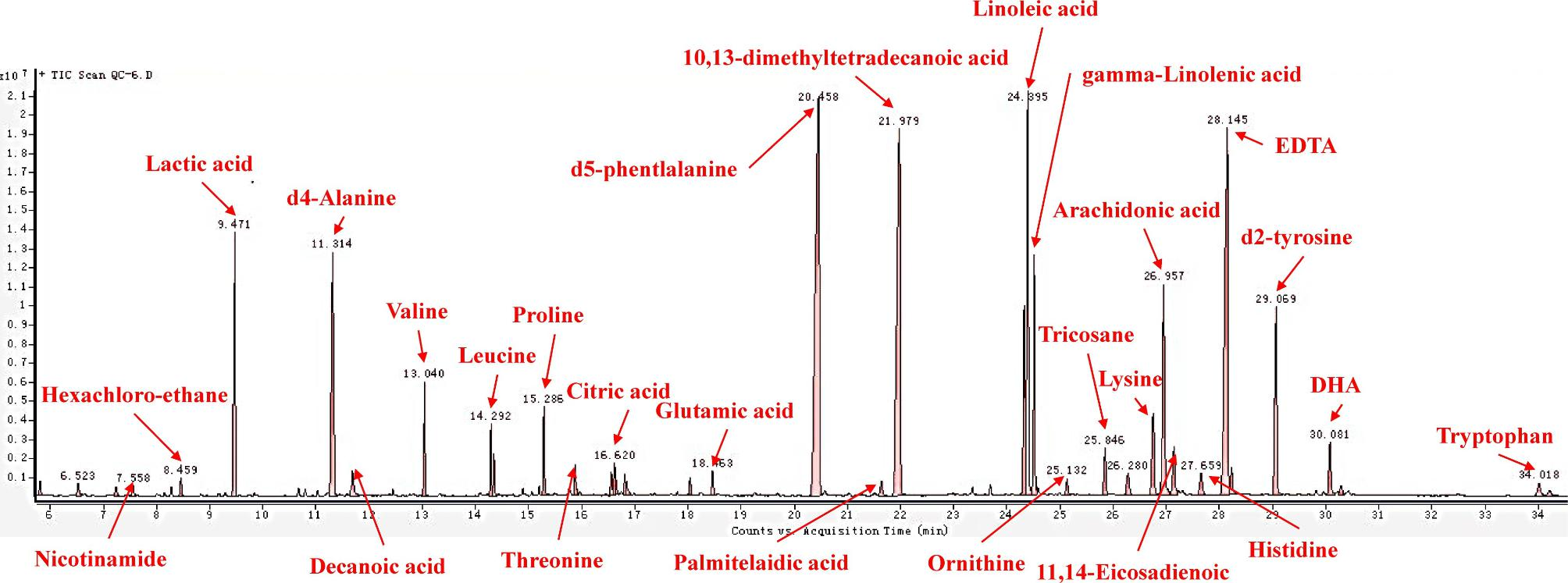

Supplement: Supplementary file 3 — Supplementary Material 3 Fig. 2: Representative total ion chromatogram (TIC) of the umbilical cord plasma metabolome [file 12986_2023_744_MOESM3_ESM.png]

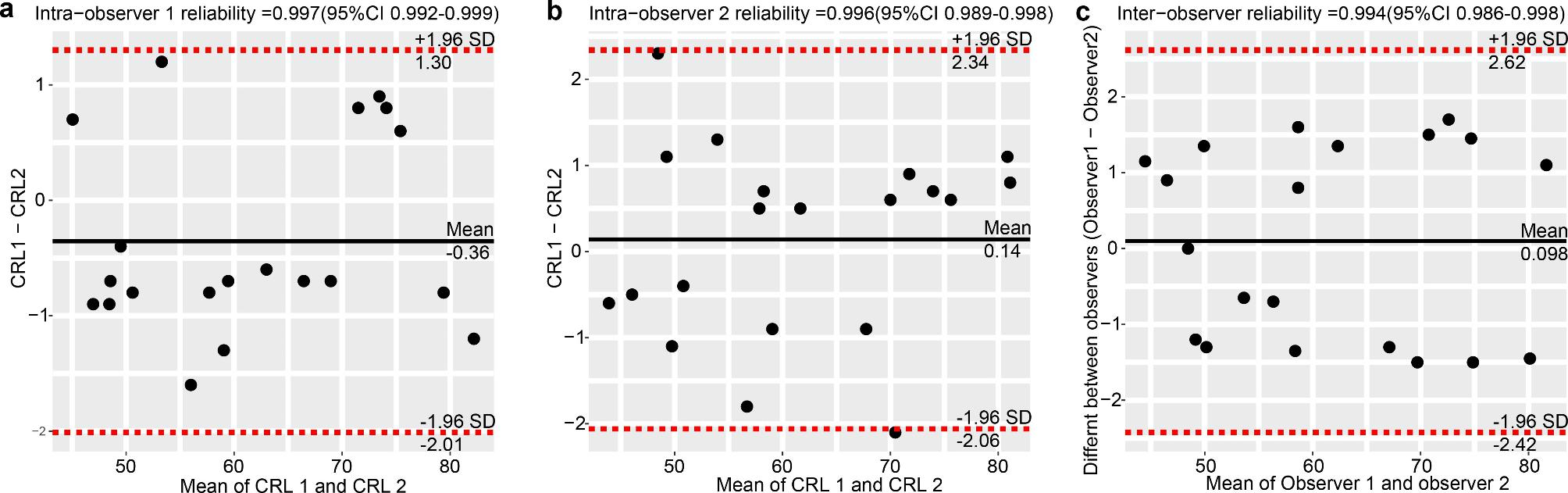

Supplement: Supplementary file 4 — Supplementary Material 4 Fig. 3: Intra-observer 1 variability (a), intra-observer 2 variability (b), and inter-observer variability (c) for determining gestational age. The proportional difference from the average for the crown?rump lengths (CRL) of 20 larger twins were diagnosed by two independent registered sonographers (observer 1 and 2). The upper and lower red dot lines mean the 2.5th and 97.5th percentiles for limits of agreement. The middle black line is the mean [file 12986_2023_744_MOESM4_ESM.png]
